# Sclerosing odontogenic carcinoma — review of all published cases: is it a justifiable addition as a malignancy?

**DOI:** 10.1016/j.bjorl.2021.01.007

**Published:** 2021-02-27

**Authors:** Daniel Lim, Chuey Chuan Tan, Wanninayake Mudiyanselage Tilakaratne, Yet Ching Goh

**Affiliations:** University of Malaya, Faculty of Dentistry, Department of Oral and Maxillofacial Clinical Sciences, Malaysia

**Keywords:** Sclerosing odontogenic carcinoma, Review, Malignancy

## Abstract

**Introduction:**

Sclerosing odontogenic carcinoma was a new addition to the list of head and neck tumors by World Health Organization in 2017. This lesion has scarcely been reported and a lack of pathognomonic markers for diagnosis exists.

**Objective:**

The aim of the study was to summarize findings from the available literature to provide up-to-date information on sclerosing odontogenic carcinoma and to analyse clinical, radiological, and histopathological features to obtain information for and against as an odontogenic malignancy.

**Methods:**

We conducted a comprehensive review of literature by searching Pubmed, EBSCO and Web of Science databases, according to PRISMA guidelines. All the cases reported as sclerosing odontogenic carcinoma in English were included. Data retrieved from the articles were gender, age, clinical features, site, relevant medical history, radiographical findings, histopathological findings, immunohistochemical findings, treatments provided and prognosis.

**Results:**

Mean age at diagnosis of sclerosing odontogenic carcinoma was 54.4 years with a very slight female predilection. Sclerosing odontogenic carcinoma was commonly reported in the mandible as an expansile swelling which can be asymptomatic or associated with pain or paraesthesia. They appeared radiolucent with cortical resorption in radiograph evaluation. Histologically, sclerosing odontogenic carcinoma was composed of epithelioid cells in dense, fibrous, or sclerotic stroma with equivocal perineural invasion. Mild cellular atypia and inconspicuous mitotic activity were observed. There is no specific immunohistochemical marker for sclerosing odontogenic carcinoma. AE1/AE3, CK 5/6, CK 14, CK19, p63 and E-cadherin were the widely expressed markers for sclerosing odontogenic carcinoma. Surgical resection was the main treatment provided with no recurrence in most cases. No cases of metastasis were reported.

**Conclusion:**

From the literature available, sclerosing odontogenic carcinoma is justifiable as a malignant tumor with no or unknown metastatic potential which can be adequately treated with surgical resection. However, there is insufficient evidence for histological grading or degree of malignancy of this tumor.

## Introduction

Sclerosing odontogenic carcinoma (SOC) was one of the new additions to the list of head and neck tumors by the World Health Organization^1^ in 2017 and is defined as a primary intraosseous carcinoma of the jaws, with bland cytology, markedly sclerotic stroma and an aggressive infiltrative pattern. Although the first report of 3 cases was published in 2008 by Koutlas et al.,^2^ this terminology was first coined by Landwehr and Allen^3^ in 1996 when they presented their case in the 50th Annual Meeting of the American Academy of Oral and Maxillofacial Pathology. Koutlas et al.^4^ presented 2 cases of SOC in the same meeting 9 years later. Following the first reported case, only a handful of cases have been reported to date. The scarcity of cases may be due to under-recognizing of the lesion or its description as other entities since it resembles many other tumors, such as metastatic carcinoma, epithelium-rich central odontogenic fibroma, calcifying epithelial odontogenic tumor, primary intraosseous carcinoma, clear cell odontogenic carcinoma and desmoplastic ameloblastoma.^1^ This paper aims to provide a summary of all reported cases of SOC and to analyse its features for and against its consideration as a malignant entity.

## Materials and methods

This integrative review was performed with the intention of summarizing findings from all the available literature to provide up-to-date information on SOC and to analyse its features to determine if it is justifiable to classify it as a malignancy. The PubMed, EBSCO, and Web of Science (WoS) databases were used to search for relevant articles. The search was conducted using “sclerosing odontogenic carcinoma”, “odontogenic sclerotic carcinoma” and “sclerotic odontogenic carcinoma” as keywords with the Boolean operator OR applied between the keywords. This review included all case reports in the English language found in the databases. No limit was imposed on the publication year. Both titles and abstracts were assessed to select relevant articles. Reference lists of the selected articles were also checked to trace any additional articles not found in PubMed, EBSCO and WoS. Full-text articles were then retrieved through online databases or hand searches. The data retrieved from the articles included sex, age, clinical features, site, relevant medical history, radiographical findings, histopathological findings, immunohistochemical findings, treatments provided and prognosis.

## Results

**Figure 1:**
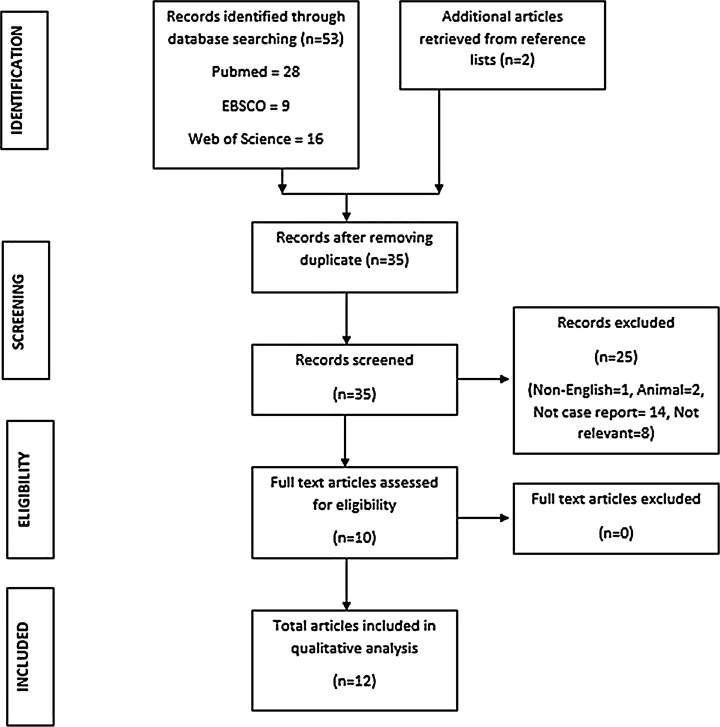
Flow diagram of literature search and identification of articles on sclerosing odontogenic carcinoma.

The literature search was performed on the 9th May 2020. A total of 53 titles were retrieved from the databases (16 articles from WoS, 9 articles from EBSCO, 28 articles from PubMed). After removing duplicates, 35 abstracts were selected for screening. In the process of identifying relevant articles, several citations were excluded. These citations included non-English articles (n = 1), case reports in animals (n = 2), articles that were not case reports (n = 14) and non-relevant articles (n = 8). Following exclusion, 10 full-text articles were assessed and included in the review. An additional 2 articles were retrieved from the reference search. In total, 12 articles containing 14 cases of SOC were included for review (Fig. 1).

According to the data, a very slight female predilection was observed. Eight cases were reported in females, while the remaining 6 cases were reported in males. The ages of patients diagnosed with SOC ranged from 31 to 73 years old, with a mean age of 54.4 years. The majority of the cases occurred during the 5th and 7th decades, with a female preponderance in the 5thdecade, while males predominated in the 7th decade. The mandible appeared to be the preferred site compared with the maxilla, as 9 cases were found in the lower jaw. Out of the 9 cases, 7 were reported in the posterior mandible, while only 2 cases occurred in the anterior mandible. Among the maxillary cases, 3 tumors were reported in the anterior maxilla, while 2 were reported in the posterior maxilla.

In most of these cases, the medical history was not remarkable except for one patient who had a history of hepatocellular carcinoma and another who had undergone surgery for breast reduction. The patients typically presented with swelling or a lump that grew progressively and rapidly. Pain was not always a symptom of this tumor, as there were cases that were asymptomatic. The tumors affecting the mandible, especially the posterior region, and can present with paraesthesia, as seen in 3 cases. Depending on the degree of bone destruction, the teeth in and around the tumor may develop mobility. A summary of the clinical features of the reported cases is shown in Table 1.

**Table 1:** Clinical characteristics of reported cases of sclerosing odontogenic carcinoma.

Authors, year	Nº of case	Age, gender	Clinical presentation	Medical Hx	Site	Initial diagnosis	Mx	Follow up
Seyiti et al., 2020^5^	1	54, Female	Firm progressive swelling, paresthesia lower lip, pain	N/A	Left posterior mandible	“Garrè’s osteomyelitis”	Resection & fibula flap	N/A
O’ Connor et al., 2019^6^	1	43, Female	Cleft between 12 and 13, asymptomatic	N/A	Anterior maxilla	SOC	Initial treatment — enucleation, followed by resection (maxillectomy)	No recurrence; 12-months
							No chemo/radio	
Todorovic et al., 2019^7^	1	62, Male	Progressive swelling, loosening of teeth, recurrent sinus infection	Hepato-cellular carcinoma	Left Maxilla	Benign fibro-osseous lesion	Left maxillectomy + removal of infratemporal fossa and skull base	Recurrence 5-months after resection.
							Recurrence — radiotherapy (66 Gy/33 fractions)	No recurrence 9-months after radiotherapy.
Kataoka et al., 2018^8^	1	68, Female	Rapid swelling of anterior mandible, no paresthesia	N/A	Anterior mandible	Benign epithelial odontogenic tumor	Resection	No recurrence; 5-years
Hanisch et al., 2017^9^	1	60, Male	Swelling in the premolar/molar region of the left mandible, no paresthesia	N/A	Left mandible	Low grade SCC	Left hemimandibulectomy, ipsilateral radical neck dissection	No recurrence; 9-months
Wood et al., 2016^10^	1	43, Female	Asymptomatic firm lump	Right breast reduction	Right anterior hard palate	Adenocarcinoma NOS	Maxillectomy	No recurrence; 17-months
Tan et al., 2014^11^	1	31, Female	Asymptomatic lump	N/A	Anterior mandible	Not mentioned	Enucleation	No recurrence; 1-year
Saxena et al., 2013^12^	1	42, Male	Firm to bony-hard swelling at left parasymphyseal area, no paresthesia	N/A	Left mandible	Epithelium-rich variant of central odontogenic fibroma	Hemimandibulectomy, radical neck dissection, radiotherapy	No recurrence; 10-months
Hussain et al., 2013^13^	1	54, Male	Firm expansile swelling	N/A	Anterior maxilla	Squamous cell carcinoma, probably of metastatic origin	Resection	No recurrence; 19-months
Irié et al., 2010^14^	1	67, Male	Paresthesia in the left lower lip and skin of the mental region	N/A	Left mandible	Benign fibro-osseous lesion	First surgery — curettage	Recurrence; 8-months after curettage.
							Recurrence — left segmental mandibulectomy, chemotherapy	No recurrence; 15-months after resection
Ide et al., 2009^15^	1	47, Female	2-cm mass on left lower lingual gingiva	N/A	Left mandible	Primary intraosseous odontogenic carcinoma with dentinoid	Resection, cervical lymph node dissection	No recurrence; 6-years
Koutlas et al., 2008^2^	3	72, Male	Enlarging left mandibular mass and mental nerve paresthesia	N/A	Left mandible	SOC	Resection and neck dissection	No recurrence; 5-years
		46, Female	Painful right mandibular lesion	N/A	Right mandible	SOC	Resection	No recurrence; 12-years
		73, Female	Enlargement of maxilla	N/A	Right maxilla	Type 3 poorly differentiated SCC	Resection and radiotherapy	No recurrence; 3.5-years

In general, this tumor presents as a radiolucent lesion in the radiograph. However, it was reported to have a ground glass appearance in one of the cases. The margins can appear well-defined, ill-defined or a combination of both. It was noted that two cases in the maxilla involved the adjacent structures, such as the nasal cavity, maxillary sinus, zygoma and pterygoid plates. Resorption of at least one of the buccal/labial or palatal/lingual cortices was reported in all cases. Resorption of dental roots and loss of lamina dura were reported in 3 cases, while there was no such resorption in 2 other cases. The remaining cases did not mention the effects on dental roots. The radiographic characteristics of the reported cases are shown in Table 2. Among the reported cases, only 2 cases were diagnosed as SOC from the beginning. Most of the cases were given other provisional diagnoses, such as Garre’s osteomyelitis, benign fibro-osseous lesion, benign epithelial odontogenic tumor, squamous cell carcinoma, adenocarcinoma, metastatic tumor, clear cell odontogenic carcinoma and primary intraosseous carcinoma.

**Table 2:** Radiographical findings of reported cases of sclerosing odontogenic carcinoma.

Authors, year	R/G findings				
	Radiopacity	Borders	Cortex	Internal	Adjacent teeth
Seyiti et al., 2020^5^	Radiolucent	Irregular	Buccal and lingual resorption, periosteum osteogenesis	Patchy calcifications	Loss of periodontal ligament and lamina dura
O’ Connor et al., 2019^6^	Radiolucent	Well-demarcated, extend to floor of nose and maxillary sinus	Buccal and palatal resorption	NIL	Erosion of roots
Todorovic et al., 2019^7^	Ground glass appearance	Irregular, involving zygoma, pterygoid, floor of orbit, nasal cavity, maxillary sinus	Buccal and palatal resorption	Loss of trabeculation	NIL
Kataoka et al., 2018^8^	Radiolucent	Well-defined	Lingual cortex intact, labial cortex resorption	Heterogenous circular mass (MRI)	No resorption
Hanisch et al., 2017^9^	Radiolucent	Ill-defined	Expansion, erosion, and perforation	NIL	NIL
Wood et al., 2016^10^	Soft tissue mass	NIL	No bony destruction	NIL	NIL
Tan et al., 2014^11^	Radiolucent	Well-defined	NIL	Specks of radiopacities	NIL
Saxena et al., 2013^12^	Radiolucent	Well-defined	Buccal and lingual erosion and perforation	NIL	NIL
Hussain et al., 2013^13^	Radiolucent	Well-defined	NIL	NIL	Loss of lamina dura and root resorption
Irié et al., 2010^14^	Radiolucent	Well and ill-defined	Resorption of buccal cortex	Mixed radiopacities and radiolucencies	No resorption
Ide et al., 2009^15^	Radiolucent	Sclerotic inferior border	NIL	NIL	NIL
Koutlas et al., 2008^2^	NIL	NIL	NIL	NIL	NIL
	Radiolucent	Ill-defined	Perforation of buccal cortex, thinning of lingual cortex	NIL	NIL
	NIL	NIL	NIl	NIL	NIL

Histopathologically, the neoplastic cells were composed of single-file thin cords, strands, and islands of epithelioid cells. There was only one case documented with a fibrous capsule encasing the tumor. The other cases were reported with an infiltrative tumor growth pattern and a lack of encapsulation. Generally, a mild degree of cellular atypia was observed in 10 cases. A dense, fibrous, collagenous, or sclerotic stromal component was the hallmark feature seen in 12 cases. Perineural or intraneural invasion was reported in 8 cases, while 2 cases reported no evidence of perineural invasion. Lymphovascular invasion was observed in 2 cases. The mitotic activity reported was inconspicuous or low in most cases. Necrosis was evident in 1 out of the 14 cases reported. Clear cell differentiation was observed in 5 cases, and further tests demonstrated the absence of mucin in these cells. Glandular differentiation was reported in 3 cases. Calcified tissue consisting of osseous, cementoid or dentinoid components was documented in 7 cases. A summary of the histopathological features of the reported cases is shown in Table 3.

**Table 3:** Histological findings of reported cases of sclerosing odontogenic carcinoma.

Author, Year	Encapsulation	Cellular atypia	Stroma	Perineural invasion	Lympho-vascular invasion	Mitotic activity	Necrosis	Clear cell differentiation	Glandular differentiation	Calcified materials
Seyiti et al., 2020^5^	NR	NR	Fibrous	Present	NR	NR	NR	NR	NR	Osseous trabeculae
O’ Connor et al, 2019^6^	NR	Mild	Collagenous and sclerosed	Present	NR	No	NR	Present (mucin negative)	NR	NR
Todorovic et al., 2019^7^	Absent	Mild-moderate	Dense fibroblastic	Absent	Absent	Low	Absent	NR	NR	Irregular trabeculae of woven bone
Kataoka et al., 2018^8^	Present	Mild	Sclerosing fibrous	Absent	Absent	Absent	Absent	NR	NR	NR
Hanisch et al., 2017^9^	NR	NR	NR	Present	NR	NR	NR	NR	NR	NR
Wood et al., 2016^10^	NR	Mild	Sclerosed collagen bundles	Present	Absent	Low	NR	“Signet-ring” appearing cells (mucin negative)	NR	NR
Tan et al., 2014^11^	Absent	Mild	Sclerotic stroma	NR	NR	Low	Absent	NR	Absent	Reactive bone
Saxena et al., 2013^12^	NR	Dysplastic	Densely collagenized	Present	Present	Low	Present	NR	Present	Small round to ovoid calcifications
Hussain et al., 2013^13^	Absent	Mild	Dense and sclerotic	Present	Present	Inconspicuous	NR	Present	NR	NR
Irié et al., 2010^14^	NR	Inconspicuous	Fibrous	Present	NR	Inconspicuous	NR	NR	Present	Cementum-like ossicles and trabecular bone tissues with or without osteoblast rimming
Ide et al., 2009^15^	NR	Mild	NR	NR	NR	Low	NR	Present (mucin negative)	Present	Dentinoid
Koutlas et al., 2008^2^	NR	Present	Diffuse sclerosis	Present	NR	Rare	NR	Present (mucin negative)	NR	Small round to ovoid acellular calcifications

NR, not reported.

Special staining with Congo red was performed in 2 cases and yielded negative findings. Immunohistochemically, AE1/AE3 or pan-CK, CK5/6, CK14, CK19, p63 and E-cadherin were the widely expressed markers. CK8/18 and CK7 were variably expressed in the reported cases. Ki-67 staining showed a low proliferative index in 6 cases. Immunostaining for p16, p40, p53, EMA and PR was reported to be positive in a single case each. Immunostaining with CEA, ER, PAX8, SMA, CD34, desmin, S-100 protein, calretinin, vimentin, amelogenin and CD1a yielded negative findings. Molecular studies for EWSR1 rearrangement were negative in 5 cases. Table 4 summarizes the immunohistochemical findings of the reported cases.

**Table 4:** Immunohistochemical findings of reported cases of sclerosing odontogenic carcinoma.

Authors, Year	Seyiti et al., 2020 ^5^	O’ Connor, 2019^6^	Todorovic et al., 2019^7^	Kataoka et al., 2018^8^	Hanisch et al., 2017^9^	Wood et al., 2016 ^10^	Tan et al., 2014 ^11^	Saxena et al., 2013 ^12^	Hussain et al., 2013 ^13^	Irié et al., 2010 ^14^	Ide et al., 2009 ^15^	Koutlas et al., 2008^2^
Special stain												
Congo red	NR	NR	NR	NR	NR	NR	–	NR	–	NR	NR	NR
IHC												
CK	NR	+	NR	+	+	+ (weak)	NR	NR	+	+	NR	NR
CK5/6	+	+	+	–	+	NR	+	+	+	+ (CK6)	NR	+
CK7	NR	–	–	–	NR	NR	+	NR	NR	+ (focal)	NR	+ (focal)
CK8/18	NR	NR	NR	NR	NR	NR	+	NR	NR	– (CK8)	NR	–
CK14	NR	+	+	NR	NR	+	NR	NR	NR	NR	NR	NR
CK19	NR	+	–	+	NR	+	+	NR	+	+	NR	+
CK20	NR	NR	–	NR	NR	NR	–	NR	NR	–	NR	–
P16	NR	NR	NR	NR	NR	NR	+ (weak)	NR	NR	NR	NR	NR
P40	NR	NR	NR	NR	+	NR	NR	NR	NR	NR	NR	NR
P53	NR	NR	NR	NR	NR	NR	+	NR	NR	NR	NR	NR
P63	+	NR	+	+	+	+ (weak)	+	+	NR	+	NR	+
CEA	NR	NR	NR	NR	NR	NR	–	NR	NR	–	NR	–
E-cadherin	NR	NR	NR	NR	NR	+	+	NR	NR	NR	NR	+
EMA	NR	NR	NR	+	NR	NR	–	NR	NR	NR	NR	NR
Ki-67	10%	<1%	10%	2%	NR	NR	<2%	NR	NR	<3%	NR	NR
ER	NR	NR	–	NR	NR	NR	–	NR	NR	NR	NR	NR
PR/PgR	NR	NR	NR	NR	NR	–	– (weak)	NR	NR	NR	NR	NR
PAX8	NR	NR	–	NR	NR	NR	NR	NR	NR	NR	NR	NR
SMA	–	NR	NR	NR	NR	NR	–	–	NR	–	NR	–
CD34	NR	NR	NR	NR	NR	NR	–	NR	NR	–	NR	NR
Desmin	–	NR	NR	NR	NR	NR	–	–	NR	NR	NR	–
S-100	–	NR	NR	NR	NR	–	–	–	NR	–	NR	–
Calretinin	NR	NR	NR	NR	NR	NR	–	NR	NR	–	NR	NR
Vimentin	NR	NR	NR	NR	NR	NR	–	NR	NR	–	NR	NR
Amelogenin	NR	NR	NR	NR	NR	NR	NR	NR	NR	–	NR	NR
CD1a	NR	NR	NR	NR	NR	NR	–	NR	NR	NR	NR	NR

ISH												
EBER	NR	NR	–	NR	NR	NR	NR	NR	NR	NR	NR	NR

FISH												
EWSR 1	–	–	–	NR	NR	–	–	NR	NR	NR	NR	NR

NR, not reported; (+), positive; (−), negative.

Radical surgery was the preferred treatment modality. These surgeries included resection, hemimandibulectomy and maxillectomy. Concurrent neck dissections were performed in 4 cases. Adjunctive radiotherapy was used in 2 cases, while recurrence was reported in 2 cases. Recurrence occurred relatively early following the first surgery. One recurred after 5 months, while another recurred at the 8th month post-surgery. The former was an extensive tumor in which left maxillectomy and removal of the infratemporal fossa and the base of the skull were performed. Following recurrence, radiotherapy (66 Gy for 33 fractions) was administered. No recurrence was observed after 9 months of followup. In another case, the tumor was initially treated with curettage. Segmental hemimandibulectomy as a second surgery and chemotherapy were performed; the patient remained tumor-free for 15 months after the second surgery. No recurrence was observed in the rest of the cases, including one that was treated only by enucleation. The followup periods ranged from 9 months to 12 years. Metastasis was not reported in any of the cases.

## Discussion

Despite being a controversial lesion, in 2017 the WHO included SOC as a new odontogenic tumor of the head and neck, although some were not convinced that it was a new entity.^16^ Whether SOC is a distinct entity or merely a different histological pattern is a concern. However, the WHO was of the opinion that SOC merits recognition as a separate entity.^1^ However, the definition of SOC by the WHO is controversial in the context of primary intraosseous carcinoma, as the latter is a separate and distinctive entity included in the category of odontogenic carcinomas. The current definition would mislead readers that SOC is one of the variants of primary intraosseous carcinoma. It should be labelled an odontogenic carcinoma rather than a primary intraosseous carcinoma, which is a definable separate entity in the classification. The diagnosis of SOC is challenging, as it shares some features with other tumors, such as odontogenic fibroma, desmoplastic ameloblastoma, calcifying epithelial odontogenic tumor, primary intraosseous carcinoma, clear cell odontogenic carcinoma and certain metastatic tumors to the jaw.^1^, ^17^, ^18^ The above differential diagnosis of SOC suggests that it may have a wide spectrum in terms of malignancy grading. The characteristics of these possible differential diagnoses are summarized in Table 5.

**Table 5:** Characteristics of differential diagnoses for sclerosing odontogenic carcinoma.

Differential diagnosis	Age	Gender	Site	Radiographic features	Histology features	Hard tissue	Management	Distinguishable features
Epithelium-rich central odontogenic fibroma^1^	Wide patient age range	Female > male	Maxilla lesion: anterior to first molar	Well-defined unilocular radiolucency	Moderately cellular or collagenous connective tissue with inactive-looking odontogenic epithelial islands or strands.	Hard tissue may present (Dentinoid or cementum like calcification)	Enucleation and curettage	Lack of infiltrative features compared to SOC
			Mandibular lesion: Posterior to first molar	Corticated margins				The stroma of SOC is sclerosed whereas COF’s stroma is variable cellularity fibrous tissue.
Calcifying epithelial odontogenic tumor^1^	Mean age = 40	Male > female	Mandible > maxilla	Mixed radiopaque and radiolucent lesion	Polyhedral neoplastic epithelial cells in islands, cords, or trabeculae.	Liesegang rings	Local surgical removal	CEOT has homogenous masses of eosinophilic hyaline material that stain positively for amyloid
				Well defined border	Pleomorphic nuclei, giant nuclei, and low mitotic rate.			
Desmoplastic ameloblastoma^1^	4th to 5th decades of life	Male = female	Maxilla > mandible	Mixed radiolucent and radiopaque appearance	Cuboidal to flat peripheral cells with central spindle-shaped cells and densely collagenous	Metaplastic bone	Wide surgical excision	MAPK pathway mutations in ameloblastoma
			Anterior region of jaw					Palisaded peripheral pre-ameloblast like cells in ameloblastoma is not seen in SOC
Primary intraosseous carcinoma, NOS^1^	Mean age = 55–60	Male > female	Mandible > maxilla	Poorly defined, non-corticated radiolucency	Islands or small nests of neoplastic squamous epithelium	–	Radical resection	Diagnosis by exclusion
								Stroma component is not sclerosed as SOC
Clear cell odontogenic carcinoma^1^	Mean age = 53	Female > male	Mandible > maxilla	Poorly defined radiolucency	Clear cells and peripheral basaloid cells in lobular sheets, islands, trabeculae, or strands	Dentinoid is present in 7% of cases	Surgical resection	Diagnosis by exclusion
								Clear cell change is not a prominent feature in SOC
Metastasis to jaws^18^	50–60 years old	Male > female	Mandible > maxilla	Poorly defined osteolytic lesions	Histomorphologically resemblance the primary tumor	–	Surgical resection	Diagnosis by exclusion
					Most common tumor site — male: lung; female: breast			Requires correlation of clinical, radiological and immunohistochemical findings for diagnosis making
					Requires immunohistochemical study to confirm the origin			

The clinical features reported are in the spectrum of indolent to aggressive behavior, causing difficulty in deciding whether a malignancy is present in the clinical settings of some of the cases. Histopathologically, SOC classically presents as cytologically bland epithelial cells within the sclerotic stroma.^1^ These two features are distinctive features for the diagnosis of a benign tumor rather than a malignancy. From the reported cases, the degree of cellular atypia was mild, and mitotic activity was generally absent or low. It remains controversial whether to label inconspicuous cellular and nuclear pleomorphism and equivocal mitotic activity as indicative of a malignancy for SOC. It has been documented that perineural invasion is a characteristic feature of SOC.^1^ This feature, however, was reported in 8 cases, while the absence of perineural invasion was reported in the remaining cases. The presence of perineural invasion is a recognized histopathological prognosticator in oral and oropharyngeal squamous cell carcinoma.^19^, ^20^ Perineural invasion is a common histopathological finding for high-grade malignancy and confers poor clinical outcomes, regional recurrence, distant metastasis and mortality.^19^ More cases are needed to evaluate whether perineural invasion is a characteristic histopathological feature of SOC and to validate it as a feature for grading the degree of malignancy.

Five cases reported a clear cell population in SOC, and further tests confirmed the presence of intracytoplasmic glycogen. This finding supports the odontogenic origin of SOC, as remnants of dental lamina and rests of Malassez would give rise to the clear cell appearance in the lesional tissue.^21^ Interestingly, a hard tissue component was observed in half of the cases reported. The association between calcifications similar to benign fibro-osseous lesions and the presence of dental hard tissue has yet to be investigated. We hypothesize that dental hard tissue formation may be attributed to the odontogenic origin of SOC and its pluripotent ability to synthesize dental hard tissue.

The neoplastic epithelial cells in SOC were positively stained with pan-CK, CK5/6, CK14, CK19 and p63. Cytokeratin (CK) is a specific marker of the epithelial cell lineage. Epithelial cells express different subtypes of cytokeratin depending on the stage of development and the stage in the sequence of terminal differentiation.^22^ The available literature shows that SOC expressed mostly high molecular weight cytokeratin, namely, CK5/6 and CK14. This finding is consistent with Crivelini et al.,^23^ who documented that the typical immunohistochemical marker for odontogenic epithelium is CK14. CK19 is a low molecular weight cytokeratin and often highlights epithelial cells near the surface epithelium or squamous differentiation.^23^ This marker, however, was unexpectedly negative in the case reported by Todorovic et al.^7^ More clarifications of cytokeratin subtype staining would be beneficial to identify the expression of cytokeratin pertaining to its histogenesis. Predictive biomarkers such as Ki-67 that are widely used to gauge the proliferative index of a tumor appeared to be a non-significant finding for SOC. Hence, histopathological examination remains the gold standard in the diagnosis of SOC, as no distinctive marker besides cytokeratin has been identified to date.

EWSR1 is the most common gene that can generate various fusion genes and is evident in a variety of neoplasms. Tumors harboring EWSR1 gene rearrangements include Ewing sarcoma, myxoid liposarcoma, clear cell sarcomas and myoepithelial neoplasms.^24^ For the head and neck region, mucoepidermoid carcinoma, clear cell carcinoma and myoepithelial carcinoma have been reported to have EWSR1 gene rearrangement.^1^ Five reported cases of SOC were subjected to FISH molecular study for EWSR1 gene rearrangement and yielded negative results. This could lead to the postulation that SOC does not exhibit EWSR1 gene rearrangement.

From the available literature, it would be safe to recommend resection of the tumor with a 5 mm margin followed by close followup after the surgery.^13^ Neck dissection is not mandatory, and chemoradiotherapy may not have a curative role, as most of the patients did not undergo such procedures but remained disease-free throughout the followup period. Metastasis has not been reported thus far. This may be related to the low-grade characteristics of epithelial and stromal components, including their low mitotic activity.^7^ Saxena et al.^25^ also postulated that the dense stroma surrounding the epithelial component of the tumor may have a role in preventing metastasis. The role of adjuvant treatment cannot be justified at the moment.

## Conclusion

From these limited numbers of cases, we can summarize that SOC is a low-grade carcinoma with no metastatic potential that can be adequately treated with local resection of the tumor. However, more information is needed to determine the definite grade of malignancy. The authors are of the opinion that SOC should be defined as a separate odontogenic carcinoma rather than a primary intraosseous carcinoma.

## Conflicts of interest

The authors declare no conflicts of interest.
